# M2e-Based Universal Influenza A Vaccines

**DOI:** 10.3390/vaccines3010105

**Published:** 2015-02-13

**Authors:** Lei Deng, Ki Joon Cho, Walter Fiers, Xavier Saelens

**Affiliations:** 1 Inflammation Research Center, VIB, Technologiepark 927, B-9052 Ghent, Belgium; E-Mails: Lei.deng@dmbr.vib-ugent.be (L.D.); kijoonc@dmbr.UGent.be (K.C.); walter.fiers@skynet.be (W.F.); 2 Department for Biomedical Molecular Biology, Ghent University, Technologiepark 927, B-9052 Ghent, Belgium

**Keywords:** matrix protein 2, influenza, vaccines

## Abstract

The successful isolation of a human influenza virus in 1933 was soon followed by the first attempts to develop an influenza vaccine. Nowadays, vaccination is still the most effective method to prevent human influenza disease. However, licensed influenza vaccines offer protection against antigenically matching viruses, and the composition of these vaccines needs to be updated nearly every year. Vaccines that target conserved epitopes of influenza viruses would in principle not require such updating and would probably have a considerable positive impact on global human health in case of a pandemic outbreak. The extracellular domain of Matrix 2 (M2e) protein is an evolutionarily conserved region in influenza A viruses and a promising epitope for designing a universal influenza vaccine. Here we review the seminal and recent studies that focused on M2e as a vaccine antigen. We address the mechanism of action and the clinical development of M2e-vaccines. Finally, we try to foresee how M2e-based vaccines could be implemented clinically in the future.

## 1. Introduction

Human influenza viruses are respiratory pathogens that are easily transmitted from an infected patient to another susceptible individual. The virus transmits by respiratory droplets and by contact. Seasonal flu epidemics cause an estimated 250,000 deaths worldwide each year [1]. Seasonal influenza can be fatal in the elderly and in patients with pulmonary or cardiovascular diseases [2]. Translated into absolute number of cases, the global burden of human influenza infection is huge. For example, it was estimated that in 2008 seasonal influenza viruses caused 90 million new infections worldwide in children below 5 years of age and were responsible for up to 20% of all pediatric acute lower respiratory infections [3]. The catastrophic influenza pandemic that emerged in 1917–1918 and caused the so-called Spanish flu, killed an estimated 50 million people [4,5,6]. Ninety years later the world faced another H1N1 pandemic, commonly named the “Mexican flu”, which was caused by the 2009 H1N1 pandemic virus. This virus turned out to be much milder than its ancestor that emerged at the end of the First World War. Still it was estimated that it killed more than 200,000 people during the first 12 months of its circulation [7]. Influenza epidemics also cause considerable economic burden due to absence from work and school and increased hospitalization rates. In addition, the fear of zoonotic infections with avian influenza viruses and recurrent outbreaks of highly pathogenic influenza in poultry farms, killing vast numbers of animals in a very short time, comes with a huge cost for society. Furthermore, transmission of an H7N9 virus (classified as a low pathogenic influenza virus) from poultry to human, which started to surface in February 2013, was followed by closure of poultry markets and by now (fall of 2014) has caused direct and indirect economic losses of more than 1.8 billion US dollars [8].

Influenza viruses are negative-stranded RNA viruses belonging to the *Orthomyxoviridae* family, and have a segmented genome. Influenza A, B, and C type influenza viruses can be distinguished based on antigenic differences in the internal viral proteins. Only influenza A viruses are known to cause pandemics. Apart from humans, other natural hosts of influenza A viruses are pigs, horses, dogs, and waterfowl. This implies that there is a tremendous reservoir of influenza genes in those species. Since influenza viruses can exchange gene segments by a process called reassortment (*antigenic shift*), there is a constant awareness that such an unpredictable event could cause the next pandemic [9]. Influenza A viruses can be subtyped on the basis of genetic and antigenic differences in the two major membrane glycoproteins: hemagglutinin (HA) and neuraminidase (NA). There are 18 HA subtypes and 11 NA subtypes [10]. The pathogenicity and virulence of influenza A viruses are variable. For example the highly pathogenic avian influenza are mainly H5 and H7 viruses, and human H3N2 viruses are typically more virulent than H1N1 viruses. Factors that contribute to the pathogenicity of influenza A include the level of pre-existing immunity to the virus, the receptor specificity of the viral HA, the type of proteases that enable proteolytic maturation of HA and the genetic predisposition of the host [11,12,13]. Influenza B viruses are nearly exclusively found in humans, and infection with influenza C viruses are usually asymptomatic or cause mild upper respiratory tract infection [14,15,16].

Influenza is a preventable disease and vaccination is considered the most effective way to control the spread of disease caused by influenza viruses. Licensed influenza vaccines are based on eliciting neutralizing antibodies against HA and can be grouped into three types. First and most used are injectable influenza subunit vaccines like Fluarix^®^ (GlaxoSmithKline, London, United Kingdom), Flulaval^®^ (GlaxoSmithKline), Fluzone^®^ (Sanofi Pasteur, Lyon, France), Afluria^®^ (bioCSL Pty. Ltd., Pennsylvania, PA, USA), Anflu^®^ (Sinovac Biotech Ltd., Beijing, China), Fluvirin^®^ (Novartis, Basel, Switzerland), Fluad^®^ (Novartis), Begripal^®^ (Novartis), cell-based Optaflu^®^ (Novartis), and Flucelvax^®^ (Novartis). A second type of vaccine is based on recombinant HA expression in insect cells, which is marketed as Flubloc^®^ in the USA. The third vaccine is intranasally applied and consists of live-attenuated influenza viruses [17,18]. Surveillance studies over the past 15 years have revealed that next to the circulating A/H1N1 and A/H3N2 viruses, two antigenically distinct B virus types circulate: the so called B/Yamagata and B/Victoria lineages [19,20]. One of these two B lineage viruses tends to be dominant over the other during seasonal influenza. In hindsight, the vaccine composition was mismatched with regard to the prevalent influenza B virus in half of the flu seasons between 1999 and 2012 [21]. Therefore, quadrivalent vaccines with two A strains and two B strains are now marketed in several countries. Clinical studies indicate that these quadrivalent influenza vaccines are as safe as trivalent vaccines and are expected to provide broader protection against influenza B virus infection [22,23].

The adaptive immune response that is built up in the human population due to infection with seasonal influenza viruses creates an immune selection pressure on the circulating viruses, which favors fit mutant viruses that at least partially escape the prevailing herd immunity. Those escape viruses carry mutations in the major antigenic regions of HA and NA, and this subtle immune escape mechanism is known as antigenic drift. Because of this, the composition of human influenza vaccines is changed almost yearly to try to match the newly circulating drift viruses. The decision on the influenza vaccine composition is taken by specialists from the World Health Organization (WHO)’s Influenza Collaborating Centers, based on virological surveillance. This decision is made approximately six months before the anticipated start of the influenza season in moderate climate zones. Although the accuracy of the prediction has improved over the past decade, mismatches still occur, making the vaccines less effective. Clearly, in case of 2009 influenza pandemic outbreak, this prediction approach did not work and the Mexican flu outbreak in 2009 took the influenza experts by surprise. Even though since the 1970s there is an H1N1 strain subtype present in human influenza vaccines, the HA from the pandemic A/California/04/2009 was antigenically only remotely related to the HA subtypes from all human seasonal H1N1 strains that circulated between 1977 and 2008. Instead, the HA of A/California/04/2009 was antigenically more reminiscent of the early descendants of the 1918 pandemic virus. So, not surprisingly, the conventional influenza vaccines that were ready to go in production in the spring of 2009, would have been close to futile to control the 2009 pandemic H1N1 outbreak [24]. Such a profound antigenic change in HA (sometimes accompanied by a major change in NA subtype as well) compared with HA in previously circulating seasonal influenza viruses, is named an antigenic shift.

As the epidemics of influenza A virus continue and the possible transmission of highly lethal avian influenza viruses from poultry to human remain a looming threat, vaccines that can induce broadly protective immune responses against influenza A are urgently needed. Approaches to achieve this are based on the induction of cross-reactive antibodies or T cell responses against the more conserved internal viral proteins. The aims are often to induce protection against all influenza A virus subtypes or in some cases against all virus strains from one subtype [25]. Here we focus on the development of universal influenza A vaccine candidates based on the conserved M2e. Its sequence conservation, accessibility for antibodies and the relative ease with which anti-M2e immunogenicity can be elicited have led to numerous attempts and designs of M2e-based universal influenza A vaccines, a few of which have even been evaluated in early stage clinical trials.

## 2. Biological Function of M2

The influenza A virus genome codes for 11 polypeptides and a number of minor proteins that are expressed by most viral strains [26]. Lamb *et al.* discovered M2 in influenza A viruses and showed that it was encoded by gene segment 7. M2 contains 97 amino acids and is expressed from a spliced mRNA derived from the M1 mRNA [27]. M1 and M2 share the first nine amino acids at their NH_2_-termini [28]. The principle function of M2 is to act as a viroporin. Soon after virion entry in the host cell, M2 becomes activated by the acidic environment within the endosomes and as a result M2 conducts the flux of H^+^ ions across the viral membrane into the virion interior. This proton influx loosens the interactions between the viral ribonucleoprotein complexes (vRNPs) and M1, a process that is named “priming”. M2 also allows the influx of potassium ions (K^+^) and sodium ions (Na^+^) although its permeability for K^+^ is 10^5^ to 10^6^ fold lower than for protons [29]. However, the concentration of K^+^ in late endosomes is close to 100 mM, sufficiently high for M2 to let this cation pass. The influx of K^+^ into the virion interior causes a second priming event during which the conformation of M1 is changed further and the viral ribonucleoprotein complexes become relaxed [30]. The low endosomal pH also triggers the membrane fusion activity of HA, which catalyzes the fusion of the viral envelope with the endosomal membrane. Since by that time the electrostatic interaction between M1 and vRNPs are lost, membrane fusion is accompanied by the release of the vRNPs into the cytosol [31].

M2 is a tetrameric type III membrane protein. Cysteine residues at position 17 and 19 are highly conserved and oxidized, and they presumably stabilize the tetrameric structure. The M2 protein can be divided into three parts: the extracellular N-terminal domain (M2e, positions 2–24), the transmembrane (TM) domain (positions 25–46) and the intracellular C-terminal domain (positions 47–97).

**Figure 1:**
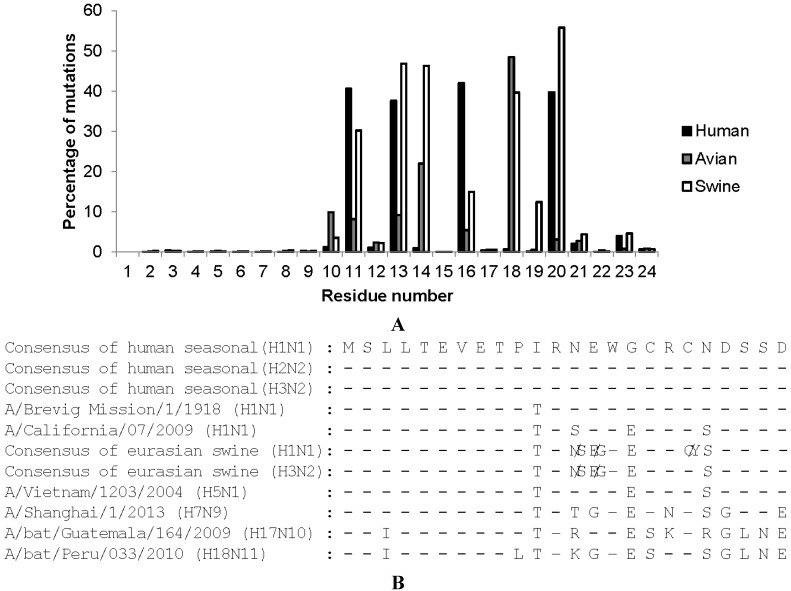
Mutation frequency of amino acids in human, avian, and swine consensus M2e. (**A**) Percentages of the residues which are different from consensus sequences were calculated based on 14,588 human, 9324 avian, and 3060 swine M2 sequences deposited in the National Center for Biotechnology Information (NCBI) databank; (**B**) Sequence alignments of M2e derived from different influenza A viruses.

The high sequence conservation of M2e among all known human influenza A viruses that circulated between 1918 and 2008, was key to its development as a universal human influenza A vaccine candidate (Figure 1A) [32]. A human influenza M2e consensus sequence was deduced, and this suggested that a human type M2 or M2e was somehow a prerequisite for influenza A viruses to be fit in the human host. However, the swine-origin H1N1 2009 pandemic virus proved this assumption wrong. This virus has avian origin gene segments 6 (encoding NA) and 7 (encoding M1/M2) and hence an “avian” type M2e, which differs at 4 positions from M2e of previously circulating human H1N1, H2N2, and H3N2 viruses (Figure 1B) [33]. The genetic relation between M2e and M1 explains the low variability in M2e. Amino acid residues 1–9 of M2e and M1 are encoded by the same nucleotides in the same reading frame. Amino acid residues 10–23 of M2e and 239–252 of M1 are also encoded by the same RNA sequence but are translated by different reading frames. A closer look at M2e shows that its N-terminal 9 amino acids are almost absolutely conserved, even in H17N10 and H18N11 influenza viruses that were recently isolated from bats (Figure 1B). M2e residues 10 to 24 are more variable. Still, in this region, Arg12, Trp15, Cys17, Cys19, and Ser22 are strongly conserved suggesting that these residues in M2e are functionally important. It is important to note that the sequence variation in the membrane proximal part of M2e is not comparable to the amino acid changes that contribute to antigenic drift in HA and NA. In the latter case, nearly any amino acid change is tolerated, whereas in the case of M2e, selection pressure is also imposed by the overlapping M1 codon sequence. An additional element that helps explain the relatively strong sequence conservation of M2e is the fact that M2e-specific antibody responses are hardly induced following an infection. Hence, there is probably only a low natural immune pressure directed against M2e [34].

Apart from M1 and M2, there are two additional mRNAs transcribed from gene segment 7: mRNA3 and −4. mRNA4 is produced by a splicing event that utilizes a suboptimal splice donor site, located in the beginning of the M1/M2 open reading frame, and the same splice acceptor site near the 3 end of the M1/M2 open reading frame. mRNA4 may give rise to an M2-like protein, named M42. M42 can functionally replace M2 *in vitro* and *in vivo* but it is non-essential for influenza A virus replication as long as M2 is adequately expressed [35]. In nature, M42 expression is probably restricted to a minority of influenza A virus strains, including human isolates and certain highly pathogenic avian influenza A virus strains [35]. Of note, the ectodomain of M42 is antigenically very different from M2. Experimental M2e-selection pressure might select for influenza A virus escape mutants that express M42, at the expense of M2.

The TM four-helix bundle drives the tetramerization and forms the pore of the low-external-pH-sensitive ion channel. Several nuclear magnetic resonance (NMR) and X-ray crystal structures of the M2 TM and of parts of the intracellular domain of M2 have been resolved. The structure at pH7.5 reveals that residues 18–23, corresponding to the extracellular membrane proximal part of M2e, and residues 47–50 are unstructured. The channel-forming TM domain (residues 25–46) forms a helix bundle with a left-handed twist angle of ~23°. Residues 51–59 form an amphipathic helix, which, in the tetramer, is oriented almost perpendicularly (~82°) to the transmembrane helices. Molecular dynamics calculation based on the crystal structure (PDB id: 3BKD) [36] suggests that the tetrameric TM domain acts as a proton transporter with Val27 and His37 functioning as a gate [37]. Sharma *et al.* proposed a mechanism for the selective proton channel function of M2. In their model, His37 and Trp41 face the inside of the tetrameric TM channel and guide protons by forming and breaking hydrogen bonds between adjacent pairs of histidines and through interactions of the histidines with the tryptophan gate [38].

Amantadine and rimantadine are adamantane-like small molecule that can block the M2 proton channel and that have been used as an antiviral drugs. There are two binding sites for amantadine that explain M2 inhibition. One is a lipid-facing pocket around Asp44 by which the drug inhibits M2 function allosterically [39,40]. The other, probably more likely an amantadine/rimantadine-binding site, is inside the pore around Ser31 by which the drug acts as a plug that blocks the M2 pore [36,41].

The M2 cytosolic tail contributes to the stable tetramer formation. This part of M2 also participates in genome packaging and facilitates virus production [42]. Deletion of the M2 cytoplasmic tail results in reduced incorporation of vRNPs into virions and strongly attenuates the influenza A virus potency. The part of M2 encompassing residues 45 to 69 in the cytosolic tail interacts with M1 protein. It has also been shown that the C-terminal 28 residues of M2 are implicated in virion budding and filamentous influenza particle assembly and release [43,44]. In particular the amphipathic alpha helix in the cytosolic domain of M2 that is close to the membrane is highly conserved and is essential for virus assembly. Recently, Rossman *et al.* showed that this amphipathic helix mediated membrane scission by altering the membrane curvature in a cholesterol-dependent manner [45]. Mutation of hydrophobic residues in this alpha helix interfere with scission and virion release. The current model for influenza A virus budding proposes that HA mediates initiation of virus budding by assembling into lipid rafts. M2 is subsequently recruited to the boundary of these lipid rafts and causes membrane scission and release of virus [45]. This procedure is the ESCRT (host endosomal sorting complex)-independent making the process of membrane scission, the final step in the budding, in case of influenza virus unique and different from that of HIV-1, Ebola virus, and paramyxovirus PIV-5.

The luminal side of the Golgi vesicles is relatively acidic. HA from most highly pathogenic H5 and H7 influenza viruses have a polybasic proteolytic maturation site that separates HA1 from HA2. As a result, the HA0 precursor from these viruses can be processed in the Golgi apparatus by furin-like proteases. Combined with the acidic environment in the Golgi vesicles, this means that HA could already convert to the post fusion state while in transit to the cell surface. M2, however, is also expressed in the Golgi and by its proton transport function, renders the Golgi less acidic. This prevents untimely conversion of HA from highly pathogenic influenza viruses into its fusogenic form [46]. The M2 proteins of different viruses vary in their ability to alter the trans-Golgi pH and the proton gating activity of M2 seems to have co-evolved with the differences in the pH that triggers membrane fusion of HA in these viruses [47,48].

M2 also influences innate host immune responses. The ion channel activity of M2 can trigger pro-inflammatory host responses by activating NLRP 3 inflammasomes in influenza A virus infected cells [49]. This activation requires M2 ion channel activity at the Golgi apparatus and operates in macrophages and dendritic cells. Autophagosome formation is an intrinsic part of eukaryotic cellular metabolism: it recycles cellular components and is implicated in the defense against intracellular pathogens. This process involves the transient formation of vesicles, called autophagosomes, that deliver cellular content such as proteins and worn-out organelles to the lysosomal compartment for degradation. Autophagy can act as an innate antiviral defense mechanism. For example herpes simplex virus-1, Kaposi’s sarcoma-associated herpesvirus and mouse herpesvirus 68, evolved to express proteins that can block the induction of autophagy, the maturation or degradation of autophagosomes [50]. Interestingly, autophagosomes accumulate after influenza A virus infection of human lung epithelial cells, but the degradation of these autophagosomes, which depends on their fusion with lysosomes, is hindered. M2 is responsible for this inhibition, independent of the ion channel activity [51]. Recently, it was found that the highly conserved M2 LC3-interacting region (LIR) close to the C-terminus of M2 bound to the LC3/ATG8 family members residing on autophagosome membrane, which mediated the redistribution of autophagosome on the plasma membrane of influenza A virus infected cells. This interaction also recruits LC3 to the plasma membrane where virion budding occurs and might mobilize lipid resources for virion budding. Mutation of M2 residues in the LIR motif dramatically reduce virus production [51,52]. Possibly, this highly conserved and functional motif in the M2 cytoplasmic tail provides a new target to develop vaccines or antivirals. However, this motif is not exposed at the cell or virion exterior.

## 3. M2-Specific Immune Responses Following Infection

M2 is a structural protein that is also abundantly expressed on the surface of infected cells. So clearly, it is exposed to the adaptive immune system of the host. Nevertheless, influenza A virus infection of humans induces a weak anti-M2 antibody response that is of short duration [34,53]. A possible explanation for this low reactivity is the small size of M2e and its low abundance of M2 in virions compared to the large glycoproteins, HA, and NA [54]. Primary infection of pigs with a high dose of H1N1 virus induced very weak anti-M2e serum IgG response. However, reinfection of such pigs with an H3N2 virus six weeks after the primary infection increased the anti-M2e IgG response by more than 10-fold [55]. A similar finding was reported in inbred and outbred mice. When mice were first infected with PR8 virus, then with PR8-Seq14 (a PR8-derived strain that evades HA-based neutralization) and thirdly with X31 virus (a H3N2 subtype virus), all animals displayed anti-M2e seroconversion. This experiment indicates that the B cell repertoire in these strains was able to generate anti-M2e antibodies [56]. From these experiments, one can conclude that M2e-specific antibody responses are poorly induced in the unprimed mammalian host, but that primary infection nevertheless elicits a degree of B cell immune memory against M2e that can be boosted by a subsequent heterosubtypic viral challenge. Experimental infection of chickens with avian influenza viruses results in a modest but detectable anti-M2e specific serum antibodies. This finding has practical implication and has been used to develop an assay to discriminate between infected and vaccinated chickens (DIVA) [57,58]. Ducks, for example, that were inoculated with inactivated avian influenza vaccine developed robust hemagglutination inhibition (HAI) titers but undetectable M2e specific antibodies [58].

Many people are frequently re-infected with influenza viruses. Zhong *et al.* analyzed the M2e-specific seroconversion during natural 2009 pandemic H1N1 infection in 118 individuals, ranging from 6 months to 53 years of age [59]. They found that the seroprevalence of anti-M2 antibodies increased with age and that a boost of this pre-existing humoral immunity against M2 was apparent following infection with pandemic H1N1 virus. In contrast, in people who had no anti-M2 antibodies before infection with the pandemic H1N1 virus, anti-M2 responses remained low in convalescent serum. The induced anti-M2 response also showed cross-reactivity with M2 from seasonal influenza A viruses [59]. It is unclear if these M2-specific antibodies contributed to protection against infection with the 2009 pandemic virus. It is also important to note that these authors used a very sensitive ELISA method that is based on reactivity of IgG against M2 expressed on mammalian cells. Under these conditions, M2 is presented as a tetramer, and hence reactivity against quaternary epitopes in M2(e) were also detectable. To summarize, following natural or experimental infection with influenza A viruses, M2e-specific antibody responses in circulation remain low. Therefore, pre-existing anti-M2e immune responses are unlikely to interfere with M2e-based vaccines.

## 4. T Cell Epitopes in M2e

**Figure 2:**
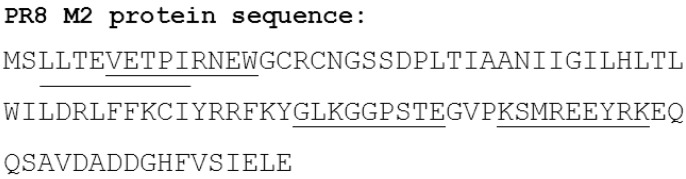
M2 from PR8 virus with the identified human T cell epitopes underlined.

M2-specific T cell responses have been described in humans [60,61]. The human CD4 and CD8 CTL epitopes directed against M2 that have been defined experimentally and are shown in Figure 2 [60,62,63]. We reported the presence of an major histocompatibility complex (MHC) class II restricted T cell epitope in M2e, that is restricted to BALB/c mouse strains (*H-2^d^)* and was strongly induced by mucosal vaccination by means of the recombinant protein CTA1-M2e-DD [64]. The CD4 T cell epitopes in M2e have been mapped for the mouse. Pejoski *et al.* found that M2e2-16 (SLLTEVETPIRNEWG) peptide contains only B cell epitopes but no T cell epitope, which explains why immunization with M2e2-16 peptide formulated in Freund’s adjuvant failed to induce antibody responses against M2e as present on virus particles. However, by including a chemically conjugated T helper epitope derived from HA in the M2e conjugate, M2e specific antibodies were readily generated [65]. In line with this, we reported that immunization with the N-terminal nine amino acids residue of M2e (SLLTEVETP) coupled to keyhole limpet haemocyanin (KLH) induced very low M2e-specific IgG responses [66].

M2e specific T cell responses varied with different host gene background. To test whether M2 vaccines showed efficacy in mouse strains other than BALB/c mice (*H-2^d^*), Wolf *et al.* elucidated that immunization with truncated M2e2-16 adjuvanted with CpG 1826 and Cholera Toxin was able to elicit a significant anti-M2e antibody response that was associated with enhanced viral clearance after X31 challenge in BALB/c (*H-2^d^*), but not in C57BL/6 (*H-2^b^*), C3H (*H-2^k^*), CD1/ICR, and Swiss Webster mouse strains [56]. The poor antibody responses in the latter mouse strains correlated with a lack of M2e-specific T cell responses in these mice [56]. The group of Dr. Epstein compared M2-specific antibody and T cell responses in BALB/c (*H-2^d^*), CBA (*H-2^k^*) and C57BL/6 (*H-2^b^*) mice after vaccination with an M2 DNA expression vector to prime the animals followed by boosting with an Adenovirus expressing M2 [67]. They reported that BALB/c mice responded strongly, CBA mice and C57BL/6 intermediately to M2 and concluded that both the MHC and background genes controled the adaptive immune response to M2. However, immunization with ASO4 adjuvanted tandem M2e-displaying lipid enveloped virus like particles (VLP), reportedly induced M2e-specific CD4 T cell responses in C57BL/6 mice as determined by ELISPOT following *in vitro* restimulation with M2e peptide [68]. Interestingly, M2-responses could be increased by adjuvanted peptide-carrier antigens in otherwise poorly responding mouse strains.

## 5. M2e-Based Vaccines

M2e has low immunogenicity in nature and is often fused with a larger carrier to enhance anti-M2e immune responses in vaccination experiments. M2e has been coupled to a plethora of carriers, usually as a genetic fusion followed by recombinant protein purification or as a synthetic peptide that is chemically linked to a suitable carrier. A very efficient and cost-effective way to make M2e immunogenic is generating recombinant virus-like particles that display M2e on their surface. Neirynck *et al.* genetically fused M2e to the hepatitis B virus core protein, which forms a virus-like particle with M2e radiating from the surface [32]. A variant of this is to use lipid enveloped VLPs in which M2e is embedded by its natural or a heterologous membrane anchor. For example, tandem repeats of M2e fused to the transmembrane domain of hemagglutinin were efficiently incorporated into VLPs and induced broad protection against influenza A virus challenge [69]. Many types of VLPs have been used to display M2e and to evaluate their efficacy as vaccines. Ease and economy of expression and purification may vary significantly, and successful approaches include the use of Malva mosaic virus (MaMV) nanoparticles [70], tobacco mosaic virus coat protein [71], potato virus X [72], Alternanthera mosaic virus coat protein [72], papaya mosaic virus [73], human papillomavirus [74], and woodchuck hepatitis virus-like particle [75]. Bacteriophage-based M2e-displaying vaccines have also been developed, which have the notable advantage that these are very easy to grow and scale up. Bacteriophages T7 and Qβ have been used to display M2e [76,77].

Efforts have also been made to present M2e as a soluble tetrameric antigen. We used a strategy based on a tetramerizing leucine zipper derived from GCN4 (General Control Non-derepressable 4). The resulting M2e-tGCN4 elicited protective immunity and, based on a competition ELISA, we obtained evidence that this approach induced tetrameric M2e-specific antibody responses in immunized BALB/c mice [78]. M2e genetically fused to a coiled-coil forming part of non-structural protein 4 of rotavirus also resulted in tetramer formation in solution and protected BALB/c mice from 3 LD_50_ PR8 challenge [79].

Synthetic M2e peptides are very soluble and can be chemically linked to different carriers. For example, scientists from the Wistar have developed the synthetic M2e-multiple antigen peptide (MAP) constructs by linking full length or truncated M2e2-16 and different Th-determinants to a lysine-rich synthetic carrier and showed M2e-based immune protection [56,80,81,82,83]. Other examples of chemical conjugates between M2e and carriers include the use of bovine serum albumin (BSA), KLH and *Neisseria meningitidis* outer membrane protein complex (OMPC), all of which are able to induce M2e-specific protective humoral immunity [66,84,85,86].

Like most other subunit vaccine antigens, immunogenicity of M2e-fusion constructs is strongly enhanced by an adjuvant. Different approaches have been followed to fuse M2e with an adjuvant. In collaboration with the group of Nils Lycke, we took advantage of the safe and potent mucosal adjuvant CTA1-DD, which is derived from Cholera toxin but lacks the pentameric B domain of this toxin. As such we generated CTA1-3M2e-DD and showed that this recombinant protein induced robust M2e-specific IgG, IgA as well as M2e-specific CD4 T cell responses after mucosal immunization [64]. The company VaxInnate developed M2e-flagelin [87,88,89] and Merck evaluated M2e fused to OMPC [85,86]. Other proteins endowed with adjuvant activity that have been used as carriers for M2e include Brucella lumazine synthase decameric carrier [90] and recombinant nanorings composed of the human Respiratory Syncytial virus (HRSV) nucleoprotein, containing bacterial RNA fragment [91]. Interestingly, immunization with the latter construct protected against both influenza and HRSV challenge in a mouse model.

DNA vaccines remain attractive because they are cheap, easy to design and physically stable. In fact, the only marketed DNA vaccine is for prophylaxtic use in the veterinary field [92]. M2(e) has been included in different DNA vaccine formats to try to induce broad immune protection against influenza A, usually alone or as one of several components, *i.e.*, next to HA and/or NP antigens [93,94,95,96,97]. In general, when M2 was used as a gene vaccine, M2e-specific antibody responses were very low. A *caveat* of full length M2 expression by genetic vaccine approaches is that the cells that express M2 from a gene vector *in vivo* are likely to die because of the ion channel activity.

Live vectors have been successfully used to induce immune responses against a diverse set of heterologous microbial pathogens in experimental settings. One attractive advantage of live vectors is their capacity to induce strong T cell responses. However, as mentioned above, immune protection by M2e-based vaccines relies primarily on M2e-specific antibodies. Several groups have explored live vectors to induce M2-specific immune responses. In many cases, full length M2 was expressed and delivered by the vector. This strategy induces very low humoral anti-M2e responses, reminiscent of the natural response to M2e. Furthermore, it is likely that antigen-presenting cells that become infected with the recombinant virus that delivers M2, will die because of the viroporin activity of M2 [98]. Still, a number of groups succeeded in showing immune protection by M2(e)-based vaccination approaches that relied on live vector delivery. Zhou *et al.* developed the recombinant chimpanzee-derived replication-defective adenovirus (AdC) vectors, AdC68, that was modified to express M2e within variable regions 1 or 4 of the Adenoviral hexon [99]. Two intramuscular doses of this vector induced strong humoral immunity in mice against a 10 LD_50_ PR8 challenge [99]. Another replication-incompetent human adenovirus-vectored influenza vaccine was engineered to express both H5 HA and four tandem copies of M2e. Intranasal administration of this adenoviral vaccine provided heterosubtypic immunity (HSI) in BALB/c mice that lasted up to twelve months [100]. Layton *et al.* introduced M2e6-13 (EVETPIRN) combined with a potential immune enhancing epitope into the genome of a recombinant attenuated *Salmonella enteritidis* strain [101]. This live bacterium was used to immunize Leghorns chicks by oral gavage. Interestingly, the vaccination resulted in M2e-specific serum IgY responses and protected the chicks against challenge with H7N2 virus but not against H5N1 virus challenge [101]. The Baxter Bioscience group reported on the use of Modified Vaccinia Ankara (MVA) to express NP, M1, M2, PB2, the stem region of HA (hlHA) and the stem region of HA with a tandem repeat of four M2e sequences (hlHA/M2e). Immunization of BALB/c mice with MVA-M2, MVA-hlHA or MVA-hlHA/M2e provided comparable and very modest protection against challenge with H5N1, H9N2 or H7N1 viruses. Only vaccination with NP-expressing MVA vectors did provide protection against challenge with these viruses [102].

Laboratory mice are widely used to demonstrate immune protection by new experimental vaccine approaches. The mouse is an important and relevant animal model for influenza because it allows obtaining proof of concept findings of new approaches and, more importantly, allows studying the mechanism of action of the experimental vaccine at hand. This can lead to surprising findings. For example, many groups have shown that broadly protective IgG antibodies that target the conserved influenza HA stalk, have *in vitro* virus-neutralizing activity. However, when it comes to *in vivo* protection, these antibodies rely heavily on antibody-dependent cytotoxic or phagocytic mechanisms as was demonstrated using Fcγ Receptor-deficient mice [103,104]. However, there are a number of *caveats* associated with the mouse model for influenza. First, mice are not natural hosts for influenza viruses. Secondly, most researchers use lethal challenge models of influenza in mice to make conclusions on immune protection or protection by antivirals. Human influenza is rarely a lethal disease. Finally, most mouse studies are done in inbred animals that are often also immunologically naïve to influenza. This contrasts with the genetic diversity in the human population and the pre-existing B and T cell memory against influenza viruses that many people carry.

Ferrets, pigs, horses, and chickens are natural hosts for influenza. In addition, non-human primates such as macaques are increasingly used for influenza virus challenge and pathology experiments. Like humans, macaques exhibit clinical symptoms, such as fever, malaise, nasal discharge, and nonproductive cough, after influenza virus infection. Moreover, the virus load can be detected in the nasal cavity and respiratory tract [105,106]. Immune protection studies in at least one of these species are essential before clinical studies can be initiated with novel influenza vaccine candidates. So, how well do M2e-based vaccines protect against influenza in speices other than mice?

M2e chemically conjugated to KLH or OMPC was very immunogenic in BALB/c mice, ferrets, and rhesus monkeys [85]. In a direct comparison of M2e-HBc with M2e-OMPC, the Merck group reported that both experimental vaccines were highly immunogenic in mice but, surprisingly, M2e-OMPC was much more immunogenic in rhesus macaques than M2e-HBc [86].

Ferrets are attractive laboratory mammalian models for studying the pathology and transmission of human influenza viruses. The respiratory tract of the ferret resembles that of the human with regard to lung physiological characteristics and the distribution of influenza virus receptors [107,108]. Immunization of ferrets with M2e-KLH or M2e-OMPC efficiently elicited M2e-specific antibodies and significantly reduced viral shedding in challenged ferrets’ lungs [85]. Pigs can be infected with swine, human, and avian viruses because they express both avian and human influenza A preferred binding receptors: N-acetylneuraminic acid-α2, 3-galactose (NeuAcα2,3Gal) and NeuAcα2,6Gal linked sialyloligosaccharides, respectively [109]. Pigs are therefore considered an important natural mixing vessel for influenza A viruses [110]. Intradermal administration of DNA vaccine encoding M2e fused to NP induced M2e-specific serum IgG in pigs but antibody levels were much higher in animals that had been vaccinated with M2e-HBc VLPs in the presence of an adjuvant. Following challenge with a swine H1N1 virus expressing M2e that differed at six positions compared to the human M2e-HBc VLP antigen [32], all animals had increased body temperature that peaked on Day 1−2 after challenge. Remarkably, pigs that had been vaccinated with the M2e-NP genetic vaccine developed exacerbated disease following challenge, possibly due to an overt or derailed T cell response. Interestingly, the pigs with the highest antibody titers against M2e (*i.e.*, those immunized with M2e-HBc VLP in the presence of an adjuvant) quickly controlled the fever, suggesting a level of clinical protection [111]. Chickens are another economically important species and moreover occasionally transmit low or highly pathogenic influenza A viruses to human. Orally administered *Salmonella*-vectored vaccine expressing M2e in combination with a potential immune-enhancing CD154 peptide protected chickens against intranasal challenge with 10^6^ 50% embryo infectious dose per bird of H7N2, a low pathogenic avian influenza virus [101]. Intranasal administration of live *Lactococcus lactis* expressing M2e on the bacterial surface and subcutaneous injection of M2e-KLH prolonged the survival time of chickens after H5N2 highly pathogenic avian influenza virus challenge [112]. Finally, immunization of chickens with tandem copies of M2e fused with maltose binding protein (MBP) induced high M2e-specific antibody responses and protected against H5N1 challenge in chicken [113].

It is advantageous to combine M2e with other influenza A antigens. Combining HA and M2e is an attractive approach for the development of broad-spectrum influenza vaccines. Immunization with a recombinant Adenoviral (rAd) vector encoding H5 HA together with four tandem copies of M2e (rAdH5/M2e) induced superior cross-protection in BALB/c mice compared to those immunized with rAd encoding H5 HA or M2e alone. Of note, one intranasal dose of rAdH5/M2e induced a significantly higher level of M2e-specific antibodies and conferred more effective protection against heterologous H1N1 in BALB/c mice as compared to that induced by rAd encoding only M2e. This shows that rAdH5/M2e induces potent and long-lasting cross-protection [100]. A DNA vaccine encoding M2e together with H1 HA induced significantly higher HA-specific CD8+ and M2e-specific T cell responses than a DNA vaccine encoding M2e or H1 HA alone and provided complete protection against H5N2 challenge in mice [95]. An overview of the above described M2e-based vaccines is provided in Table 1.

**Table 1:** Overview of M2e based vaccines.

Overview of M2e Based Vaccines					
Vaccine Type	Carriers	Copy Numbers	Antigen Type	Immunogenicity Readout in Animal Models (Administration Routes)	Reference
VLPs	HBc	1, 2, 3	human	Mice (intranasal, intraperitoneal) Pigs (intramuscular), Human	[32,111,114,115,116]
	HA(TM)	5	human, swine, avian	Mice (intramuscular)	[69]
	MaMV	3	canine	mice (subcutaneous), dogs (intramuscular)	[70]
	Tobacco mosaic virus coat protein	1	human	Mice	[71]
	Papaya mosaic virus	1	human	Mice (subcutaneous)	[73]
	Woodchuck hepatitis VLP vectored in Salmonella Typhimurium	1	avian-like	Mice (oral)	[75]
	T7	1	human	Mice (subcutaneous)	[76]
	Q-β	1	human	Mice (intranasal, subcutaneous)	[77]
DNA	Complete NP	1	swine	Pigs (intradermal)	[111]
	VP22, tegument protein of bovine herpesvirus-1	1	human	Mice (intramuscular)	[93]
	HA, NP (147-155)	1	human	Mice (gene gun)	[94]
	HA	1,2	human, avian	Mice (gene gun, intramuscular)	[94,95]
peptide	-	1	human	Mice (subcutaneous)	[65]
	Multiple antigen peptide	1, 4	human, avian	Mice (intranasal, subcutaneous)	[56,80,81,82,83]
protein	Influenza NP	8	-	Mice	[117]
	CTA1-DD	1, 3	human	Mice (intranasal)	[64]
	tGCN4	tetramer	human	Mice (intraperitoneal, intranasal)	[78]
	rotavirus fragment NSP4	tetramer	human	Mice (subcutaneous)	[79]
	KLH	1	human, avian	Mice (subcutaneous, intramuscular), Ferrets (intramuscular), Rabbit	[66,85]
	OMPC	1	human	Ferrets (intramuscular), Rhesus Monkey (intramuscular)	[85,86]
	RSV NP	1, 3	human	Mice (intranasal, subcutaneous)	[91]
	BLS	1, 4	human	Mice (intranasal, subcutaneous, intramuscular)	[90]
	glutathione S-transferase	1, 4, 8	human	Rabbit (subcutaneous)	[118]
	flagelin	4	human	Human (intramuscular), Mice (subcutaneous, intranasal)	[87]

## 6. Mechanisms of Protection by M2e-Based Vaccines: Universal Protection and Beyond

Understanding the mechanism of protection by M2e-based vaccines is critical for future clinical trials. Since 1998, when Zebedee and Lamb reported that the M2e-specific mouse monoclonal antibody 14C2 was able to restrict the *in vitro* replication of some strains of influenza A [119], nearly all publications on M2e-based immune protection have monitored M2e-specific antibodies and often demonstrated that IgG against M2e is essential for protection against influenza A virus challenge. This is consistent with studies showing that mice can be protected against influenza A virus challenge by injection of M2e-specific monoclonal antibodies [120,121,122,123,124] or M2e-vaccine derived immune serum [32,85,125,126]. This suggests that protection by M2e vaccination depends on antibodies, and it was speculated early on that this protection was mediated by antibody dependent NK cell activity rather than by direct virus neutralization [127]. The involvement of NK cells for immune protection by M2e-specific IgG was proposed based on NK cell depletion experiments where the mice were treated with anti-asialo-GM1 before lethal PR8 challenge. This experiment revealed that there was no protection in M2eHBc immunized mice that had been depleted of NK(T) cells [127]. However, other groups reported that NK cells were not involved in the protection by M2e vaccine, showing that mice depleted of NK cells using anti-NK1.1 or anti-asialo-GM1 showed similar protection as control groups [126]. Instead, Fcγ Receptors expressed on alveolar macrophages were identified as crucial players in anti-M2e IgG mediated immune protection [128]. Wang *et al.* found that passive transfer of a human anti-M2e monoclonal antibody failed to protect C3 knockout C57Bl/6 mice against influenza A virus challenge, implying that complement contributes to protection [129]. The types of M2e-specific IgG isotypes that are induced by immunization are somewhat predictive for the protective effectiveness in mice [26,65,130]. In general, IgG2a responses reflect a Th1 type of immune response and, for viral infections, these isotype antibodies correlate better with protection than IgG1 does [131]. We found that significantly increased levels of IgG2a/IgG1 responses and improved protection against viral challenge were obtained when M2eHBc was adjuvanted with CTA1-DD, after both intranasal and intraperitoneal administration in BALB/c mice [132]. This difference correlates with the higher affinity of IgG2a antibodies for the activating receptors FcγRI, -III and -IV, whereas mouse IgG1 only binds significantly to FcγRIII [133]. Passive transfer of the IgG1 fraction purified from polyclonal M2eHBc hyper immune mouse serum protected wild type but not FcγRIII^−/−^ mice from challenge. When M2e-specific IgG2a was present, FcγRIII^−/−^ mice were protected against challenge, suggesting compensatory interactions between IgG2a and (an)other Fc receptor(s), like FcγRIV, in these knockout mice [128].

Given the importance of Fcγ Receptors for M2e-based immunity in mice, it is somewhat puzzling that immunization with M2e conjugates also works in chickens. In birds the predominant circulating antibody is IgY, which is considered an ancestral form of mammalian IgE and IgG and is functionally most similar to IgG. IgY is recognized by the high-affinity FcY receptor (chicken lg-like receptor (CHIR)-AB1), a member of the leukocyte receptor family. CHIR-AB1 binds IgY in a similar way as human FcαRI binds IgA. The IgY receptor is expressed on chicken B cells, macrophages, monocytes and NK cells [134,135]. A truncated form of IgY that lacks an Fc tail, IgY(ΔFc), is present in ducks and appears to be a structural equivalent to F(ab')_2_ fragment of mammalian IgG [136]. Extrapolating the findings on the protective mechanism of M2e-based immunity from mice to birds would mean that only full length IgY contributes to protection.

Taken together, antibody dependent cell-mediated cytotoxicity (ADCC), complement dependent cytotoxicity (CDC) and antibody dependent cell-mediated phagocytosis (ADCP) are the main mechanisms of anti-M2e IgG protective immunity. The murine FcγR family consists of four activating receptors, FcγRI, FcγRII, FcγRIII and FcγRIV. The contribution of FcγRIV in immune protection by anti-M2e IgG remains unclear and it will be interesting to address this question in the future, by studying protection using FcγRIV-deficient mice.

Based on a large set of passive transfer experiments in laboratory mice, it seems fair to conclude that antibodies directed against M2e are essential for immune protection by M2e-vaccines. A number of groups therefore explored the use of anti-M2e IgG immunotherapy to prevent or treat disease caused by influenza A virus infections. Fu *et al.* generated four anti-M2e antibody hybridomas using standard protocols for monoclonal antibody production. Epitopes were mapped in ELISA using a series of truncated M2e peptides. This showed that the antibodies recognizing the highly conserved N-terminal sequence of M2e provided better protection in mice [124]. Human monoclonal antibodies directed against the N-terminus of M2e have been isolated from a healthy individual by screening IgG^+^ memory B cells isolated from PBMC of M2e-seropositive subjects. Among these isolated antibodies, TCN-031 and TCN-032 efficiently bind to tetrameric M2 on virions or PR8-infected cells and have a low affinity for synthetic M2e peptides. In addition, these human monoclonal antibodies protected mice against challenge with highly pathogenic H5N1 virus [123]. Theraclone, the company who developed these human monoclonal antibodies, went a step further and evaluated TCN-032 in a phase I clinical study. This study revealed that parenteral administration of this M2e-specific IgG monoclonal antibody was very well tolerated. More recently, 40 healthy adult volunteers were enrolled for a phase II study to evaluate clinical protection against experimental influenza A virus challenge. This drug study showed that daily influenza symptom scores (1–7 days) were significantly reduced in TCN-032 treated subjects compared to placebo subjects. In addition, the median viral load in the TCN-032 treated and challenged volunteers was more than two logs lower compared to the placebo treated group [137]. No difference in time to peak symptoms (at Day 3) was reported, which is consistent with the M2e antibody mechanism requiring recruitment of effector immune cells to mediate infected cell clearance. This first study in humans with an M2e-based antibody therapy should be considered as an important landmark for further clinical development of active and other passive M2e-directed immunization strategies.

Song *et al.* generated a fully human M2e-specific monoclonal antibody (Z3G1). The prophylactic and therapeutic administration of Z3G1 resulted in significant protection of mice and alleviated clinical symptoms and reduced lung pathology following pandemic H1N1 infection of monkeys. A derivative of Z3G1, called AccretaMab^®^ Z3G1, was generated that had a chimeric IgG1/IgG3 Fc region and was de-fucosylated. This modification resulted in increased ADCC and CDC activities *in vitro* using PBMC from different human donors [138].

In contrast to neutralizing antibodies elicited by seasonal influenza vaccines, M2e-specific antibodies will initiate viral clearance by binding to infected cells, implying that at least a first round of infection must take place before anti-M2e antibodies can exert their protective effect. An undeniable advantage of infection-permissive M2e vaccine over HA-matching conventional vaccine is that they allow the induction of CD8 T cell immunity. Evidently, at higher challenge doses, protection of mice after immunization with M2-HBc may therefore be less potent than that achieved with UV-inactivated HA-matched influenza A vaccines. Nevertheless, we have reported that body weight loss is completely absent in M2e-VLP immunized mice that were challenged with a dose of pandemic 2009 influenza A virus that induced severe weight loss in control mice or in mice that had been vaccinated with a mismatched whole inactivated influenza vaccine [139]. Although it is unclear if annual influenza vaccination of people is associated with reduction of cross-reactive CD8^+^ T cell responses, there is a possibility that such a response could be reduced in the long term if one starts to vaccinate children from a very early age onwards [140]. Notably, influenza vaccination of people from the age of 6 months onwards is recommended in the US [141]. It has been proposed and shown in experimental vaccination and challenge experiments in mice and ferrets, that immunization with conventional vaccines can offer full protection against a homologous challenge and, on the downside, suppresses the induction of CD8 T cell responses directed against the conserved internal viral proteins, some of which are highly conserved and have been shown to contribute to protection against heterosubtypic viruses [142,143,144]. Moreover, there is some evidence that influenza vaccination of children also affects their T cell responses against H5N1 virus [145]. We showed that, in the mouse model, M2e-based immune protection was not only protective against different influenza A virus strains, but also that CD8 T cells directed against the conserved nucleoprotein of influenza A were induced in the M2e-immune animals following primary infection. Together with the M2e-specific antibodies, these CD8 T cells protected against challenge with a secondary, heterosubtypic virus [139].

The degree of M2e epitope density on the carrier molecule is a critical factor for the induction of a strong M2e-specific immune response [114,118,146]. Multiple tandem copies of M2e in a vaccine construct could elicit higher M2e IgG titers than one copy M2e containing construct. We constructed M2eHBc particles with one (1604), two (1817) or three (1818) M2e copies fused to the N-terminus of HBc subunits. We found that vaccination with 1817 or 1818 constructs induced higher M2e-specific IgG titers and protection than 1604 [115]. Likewise, an MBP-3-M2e vaccine candidate conferred better protection than a one M2e copy containing construct against H5N1 infection in chicken [113]. To induce more universal protection against influenza A virus infection, it is advised that the M2e antigen in influenza vaccine contains M2e variants with PI-PT-LT type M2e sequences (Figure 1B). The important role of the proline residue at position 10 in M2 for recognition by some early described mouse monoclonal antibodies was demonstrated in an escape selection experiment by the group of Walter Gerhard. Chronic treatment of PR8 virus infected SCID mice with anti-M2e mouse monoclonal antibodies delayed disease progression. However, eventually M2e-escape mutants broke through in the anti-M2e IgG treated SCID mice. Only viruses with P10L and P10H substitutions in M2(e) were isolated [121]. Of note, these two replacements are usually found among the highly pathogenic avian influenza H5 and H7 viruses. The M2e-specific IgG2a monoclonal antibodies that were used for treatment failed to bind to M2 from the P10L or P10H on the surface of MDCK cells that had been infected with the escape viruses [121]. However, no escape mutants emerged after 11 consecutive passages of PR8 in BALB/c mice that had been actively vaccinated with M2e. These unpublished data indicate that M2e, though not completely invariant, is still stable under strong immune pressures.

Intranasal immunization is a preferred and logical route to control disease caused by respiratory pathogens such as influenza viruses. Not surprisingly, intranasal/mucosal immunization has often been explored for M2e based vaccines. In addition, administration of a needle-free vaccine can offer a logistic advantage in case of a pandemic outbreak, when time and trained medical personnel could be limiting. Using a highly immunogenic nanoparticle based on recombinant nucleoprotein of respiratory syncytial virus substituted with three tandem copies of M2e, Herve *et al.* compared the effectiveness of intranasal and subcutaneous vaccination in BALB/c mice. Anti-M2e IgG1 and IgG2a titers were comparable in both groups but only intranasal immunization gave rise to anti-M2e IgA responses. These IgA antibodies were present in serum and locally in the bronchoalveolar lavage fluid. Intranasally immunized mice were better protected than subcutaneously immunized mice against challenge with PR8 virus [91]. This finding is in agreement with previous findings [32,56,77,81,132]. The local anti-M2e IgA induction also played a critical role in protection, but the mechanism of anti-M2e IgA mediated protection remains unclear. However, intranasal influenza vaccines for human use suffered a setback due to reported severe side effects related to the used adjuvant [147].

## 7. Clinical Development of M2e-Based Vaccines

A few Biotech companies have evaluated M2e-containing vaccines in early phase clinical trials. The prime purpose of phase I clinical studies is to document safety and, in case of vaccines, immunogenicity. Since M2e is naturally poorly immunogenic, it was important to demonstrate that M2e-vaccines could induce strong M2e-specific antibody and potentially T cell responses. Ideally, such responses should be long lasting. VaxInnate reported on their phase I clinical trial with different doses of VAX102, a recombinant flagellin protein (a TLR5 ligand) with four tandem copies of M2e fused at the C-terminus. Doses of 0.3 and 1.0 μg VAX102 injected intramuscularly were safe and induced up to one 1 μg/mL of anti-M2e IgG in circulation. Higher doses of VAX102 were however associated with severe symptoms [87].

Researchers from Dynavax constructed a recombinant protein antigen, comprising eight copies of M2e fused to NP. This protein was covalently linked to an immunostimulatory sequence (ISS) that is owned by Dynavax and is a ligand for Toll like Receptor 9, creating N8295. N8295 was successfully evaluated in two phase 1 studies in healthy volunteers, as a standalone antigen and combined with a poorly immunogenic dose of an H5N1 vaccine. N8295 induced M2e- and NP-specific antibodies as well as NP-specific cellular responses. In addition, no serious adverse events were reported and adding N8295 to the H5N1 vaccine augmented HA-specific antibody responses to that vaccine [117].

In a randomized, double-blind, placebo-controlled phase I clinical trial of M2eHBc (ACAM-FLU-A™) sponsored by Acambis (later acquired by Sanofi Pasteur), the intramuscularly injected ACAM-FLU-A™ was well tolerated and able to generate anti-M2e seroconversion in up to 90% of healthy volunteers [116,148]. However, further clinical development of this M2e vaccine candidate as a standalone vaccine is unlikely, in part because the M2e-specific antibody titers dropped fairly rapidly over time.

A DNA vaccine that comprises an M2 expression plasmid in one of the arms was also conducted. The prime purpose of this study was to show that the use of the proprietary Vaxfectin adjuvant could improve immune responses induced by DNA vaccination in humans. The clinical study involved 103 healthy adults who received two injection of Vaxfectin adjuvanted monovalent DNA (encoding H5 HA) or trivalent DNA vaccine with plasmids encoding HA, NP and M2. M2e-specific total IgG antibody responses were observed only at the highest DNA dose and in approximately one third of the subjects [149]. As mentioned earlier, DNA vaccination with full length M2 is suboptimal to induce M2e-specific immune responses.

## 8. Conclusions and Future Perspectives

Influenza vaccine is regarded as the first prophylactic line to protect people from disease caused by influenza viruses. Influenza vaccines are very safe and usually effective, however, these conventional influenza vaccines need to be updated almost every year to anticipate circulating flu viruses that antigenically deviate from those that were prevalent in former seasons. As yet, there is no licensed broadly protective influenza vaccine that could be implemented right after the start of a new pandemic. Such a universal influenza vaccine could also be used to prevent seasonal influenza, provided that it proves to be non-inferior to the existing seasonal influenza vaccines that mainly rely on the induction of strain-specific virus neutralizing antibodies. In other words the bar is high for competing technologies.

M2 is a viroporin that plays an important role during the early stages of virus entry. In addition, recent discoveries have attributed immune-modulatory roles to M2 since it disturbs and exploits autophagy and can activate the inflammasome. Remarkably, the natural immune response against M2, and in particular against M2e, is very modest, most likely due to its low abundance on virions.

**Figure 3:**
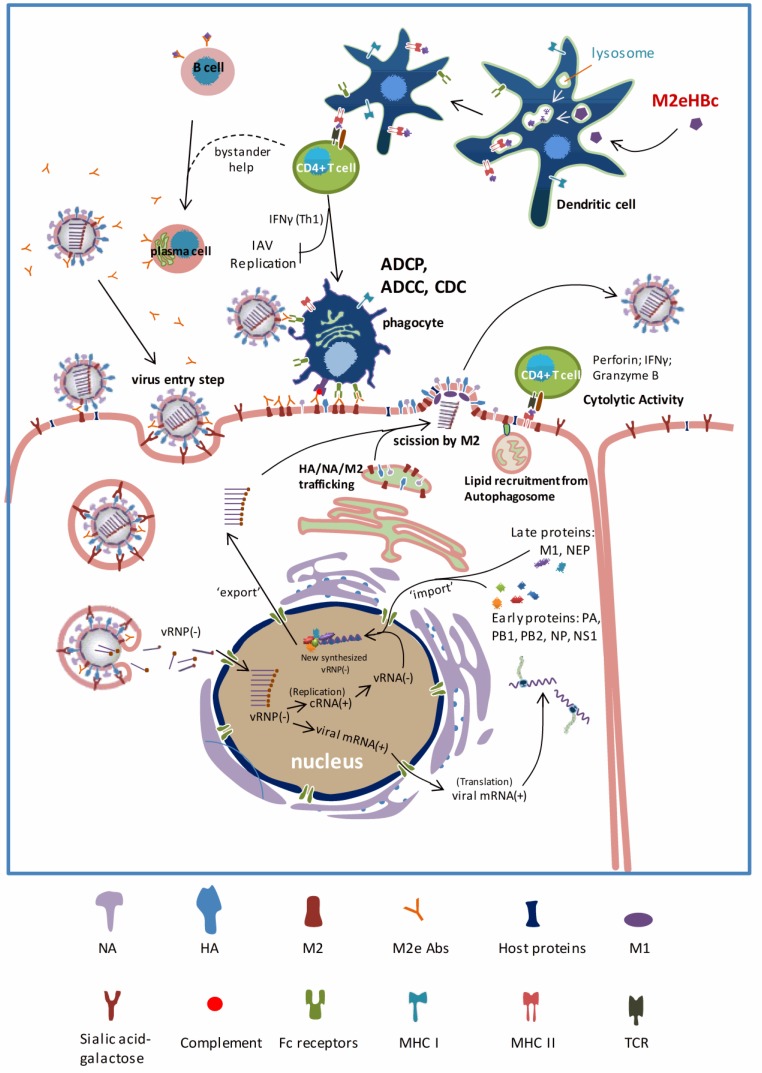
Influenza A virus infection cycle and mode of action of M2e based vaccines. The influenza A virions bind to sialic acid containing receptors on the surface of cells. Following endocytosis, the acidification of the endosome triggers the low-pH activation of M2. Then, the viral membrane fuses with the endosomal membrane by a low pH induced conformational change in HA. The interaction between M1 and vRNPs loosens after H^+^ influx by activated M2 ion channels, resulting in the release of vRNPs into the cytosol. In the nucleus, cRNA(+), vRNA(−) and mRNA(+) are produced, allowing the influenza A virus genome and proteins synthesis. Most likely M2 mediates the lipid recruitment from autophagosome during virus budding. The influenza A virus components and vRNP are packaged at the membrane, allowing the release of newly produced virions from the apical side of airway epithelial cells and the virus spreads. The critical steps in virus replication cycle and the M2(e) vaccine mechanism of action are highlighted in bold and in red. M2e-derived epitopes are presented in the context of MHC II molecules. M2e-specific CD4+ T cells are activated via T cell receptors recognition of these presented M2e epitopes, and release cytokine and chemokine in order to offer bystander help to antibody producing plasma cells or possibly clear infected cells as Cytotoxic CD4+ T lymphocytes. Phagocytes can recognize M2e-specific IgG immune complexes on the surface of infected cells and subsequently kill and eliminate the infected cell. Recognition of M2 on the surface of infected cells by phagocytic cells depends on Fc receptors and opsonizing anti-M2e IgG antibodies.

M2e is a highly conserved target for universal influenza A vaccine development. Different types of M2e-based vaccine, such as DNA vaccine, protein vaccine, VLPs vaccine, and vectored vaccine, are all able to provide a certain level of broad-spectrum protection in animal models. The influenza A virus infection cycle and possible mechanisms of M2e-based vaccine-mediated protection are summarized in Figure 3. M2e-specific antibodies, mainly IgG, are the main actors in immune protection and do so by engaging Fcγ Receptor expressing immune cells such as alveolar macrophages. It is also well documented that mucosal immunization with M2e-based vaccines offers better protection in mouse models compared to parenteral immunization strategies. This improved protection may be attributable to the induction of M2e-specific IgA. The infection-permissive character of M2e-based vaccines can be considered as an advantage when vaccinating immunologically naïve individuals. Because M2e-immunity does not neutralize the virus, the limited virus replication still induces cross-reactive T cell responses against other conserved viral antigens such as NP and M1. However, M2e will likely not be a complete substitute for the currently licensed influenza vaccines that are able to confer much stronger protection, be it against a very narrow antigenic range of viruses. In the future, with many other universal influenza vaccine candidates on the horizon, M2e-conjugate vaccines will likely find a place as part of a vaccine that is a blend of different conserved epitopes that together may offer strong, long lasting, and foremost broad immune protection. Whether such a vaccine will perform better clinically than a fully antigenic matched seasonal vaccine remains to be seen. However, such universal vaccines would prove their value in the case of a pandemic.
